# Stress Corrosion Cracking of 316L Stainless Steel Additively Manufactured with Sinter-Based Material Extrusion

**DOI:** 10.3390/ma16114006

**Published:** 2023-05-26

**Authors:** Ricardo Santamaria, Ke Wang, Mobin Salasi, Mariano Iannuzzi, Michael Y. Mendoza, Md Zakaria Quadir

**Affiliations:** 1 Curtin Corrosion Centre, Curtin University, Perth, WA 6102, Australia; ricardo.santamar@postgrad.curtin.edu.au (R.S.); ke.wang2@curtin.edu.au (K.W.); mobin.salasi@curtin.edu.au (M.S.); mariano.iannuzzi@curtin.edu.au (M.I.); 2 Institute of Naval and Maritime Science, Universidad Austral de Chile, Valdivia 5090000, Los Rios, Chile; michael.mendoza@uach.cl; 3 John de Laeter Centre, Curtin University, Perth, WA 6845, Australia

**Keywords:** chloride stress corrosion cracking (CSCC), crack-branching, C-ring specimen, porosity, residual stresses, transgranular cracking

## Abstract

This study investigates the stress corrosion cracking (SCC) behavior of type 316L stainless steel (SS316L) produced with sinter-based material extrusion additive manufacturing (AM). Sinter-based material extrusion AM produces SS316L with microstructures and mechanical properties comparable to its wrought counterpart in the annealed condition. However, despite extensive research on SCC of SS316L, little is known about the SCC of sinter-based AM SS316L. This study focuses on the influence of sintered microstructures on SCC initiation and crack-branching susceptibility. Custom-made C-rings were exposed to different stress levels in acidic chloride solutions at various temperatures. Solution-annealed (SA) and cold-drawn (CD) wrought SS316L were also tested to understand the SCC behavior of SS316L better. Results showed that sinter-based AM SS316L was more susceptible to SCC initiation than SA wrought SS316L but more resistant than CD wrought SS316L, as determined by the crack initiation time. Sinter-based AM SS316L showed a noticeably lower tendency for crack-branching than both wrought SS316L counterparts. The investigation was supported by comprehensive pre- and post-test microanalysis using light optical microscopy, scanning electron microscopy, electron backscatter diffraction, and micro-computed tomography.

## 1. Introduction 

Additive manufacturing (AM) encompasses the technologies used to create physical objects from digital data by successively joining materials [1]. Sinter-based material extrusion, one of the AM technologies categorized by the International Organization for Standardization (ISO) [2], is gaining popularity due to its ease of use, low running and maintenance costs, and reduced safety risks [3,4]. Sintered-based AM involves a multi-step approach that incorporates the principles of fused filament fabrication (FFF), also known as fused deposition modelling (FDM), powder metallurgy (PM), and metal injection molding (MIM). The process starts by heating the pre-alloyed powder-bound feedstock to the binder’s melting point, and then extruding it through a nozzle to fabricate the so-called “green part”. In the subsequent step, the primary binder is removed through full immersion in a solvent bath that leaves a component consisting of powder held by the secondary binder. This so-called “brown part” is still incomplete in terms of engineering properties. Therefore, in the final step, the component is strengthened by heating it just below the alloy’s melting point, allowing the metal particles to sinter and create a structure that requires minimal post-processing or machining. The resulting sintered microstructure has been reported to have a weak crystallographic texture, relatively large equiaxed grains, and populated with twin boundaries, pores, and oxide inclusions [5,6,7,8,9]. These characteristics diverge from the typical columnar grains found in other AM technologies where the heat follows the dissipation route, such as laser-engineered net shaping (LENS) [10,11], electron beam additive manufacturing (EBAM) [12,13], and laser-based powder bed fusion (LPBF) [14,15]. 

Stress corrosion cracking (SCC) is a form of environmentally-assisted cracking (EAC), typically nucleating from localized corrosion sites when susceptible materials are exposed above a threshold stress in specific corrosive environments [16,17,18,19,20]. For instance, in conventional austenitic stainless steels, cracks originate from pits that create the stress concentration and acidic environment required for cracking [21,22,23,24,25,26,27]. The trajectory of the crack is determined by the energy associated with its propagation process. Therefore, secondary cracks, or crack-branching, occur due to the presence of an obstacle or a more energetically favorable path [28]. The presence of a tensile stress, either residual, applied, or both, along with a specific corrodent, are required for SCC to occur. Moreover, the cracks can grow and propagate at much lower stress levels than those needed to fracture the material without the corrodent [16,18,29,30,31,32]. Therefore, brittle SCC fracture can occur on otherwise highly ductile materials [16,29,33]. 

Austenitic stainless steel UNS S31603 (SS316L) is considered an excellent material for engineering applications due to its exceptional ductility, weldability, and corrosion resistance [16,34,35]. Its low carbon content (max. 0.035% [36]) has largely eliminated sensitization of its microstructure, which is responsible for intergranular SCC [16,34,35]. However, when exposed to hot environments containing halides, stressed SS316L can still experience transgranular SCC [17,19,32,37]. Consequently, SCC poses a significant threat to the integrity and reliability of equipment in the energy sector. Thus, it is crucial to understand the influence of the AM process on SCC susceptibility. 

Among the AM technologies, there has been a significant increase in the use of LPBF to investigate SCC in SS316L due to its ability to produce an alloy with a fully austenitic microstructure [38,39,40], extremely low porosity [40,41,42], excellent resistance to localized corrosion [43,44,45,46], and outstanding mechanical properties [40,41,42]. This is due to the distinctive manufacturing process of LPBF, in which a high-intensity laser is programmed to melt layers of powder feedstock that solidify into near-net-shape parts [2,3,47] at cooling rates ranging from 10^3^ to 10^7^ K/S [48,49,50,51]. However, this heating and cooling cycle at each deposited layer results in LPBF-manufactured SS316L with high residual stresses [40,52,53], which are known to increase its susceptibility to SCC [52,54,55]. 

The objective of this work was to determine the SCC behavior of SS316L additively manufactured with sinter-based material extrusion when exposed to different stress levels and temperatures in an acidic chloride environment. The study focused on the impact of the sintered microstructure on the SCC initiation and crack-branching susceptibility. The SCC response was compared with those obtained from similarly tested wrought SS316L samples in solution-annealed (SA), and cold-drawn (CD) conditions. The investigation was supported by comprehensive pre- and post-test microanalyses that included light optical microscopy (LOM), scanning electron microscopy (SEM), electron backscattered diffraction (EBSD), and micro-computed tomography (micro-CT).

## 2. Materials and Methods

### 2.1. Materials

The AM SS316L samples used in this investigation were fabricated using Bound Metal Deposition (BMD) (Desktop Metal, Studio System; Burlington, MA, USA). The technology includes the following: (i) rods of pre-alloyed SS316L powder held in a mix of polymer and wax binder, (ii) an FDM 3D printer, (iii) a solvent-based debinding unit, (iv) a sintering furnace, and (v) a cloud-based software (Live Studio v3.0) to control the process from digital object to sintered part. Further information regarding the manufacturing process can be found in a previous publication by Santamaria, R., et al. (2021) [9]. All BMD SS316L specimens for tensile and SCC testing were produced with the parameters summarized in Table 1. For comparison, commercially available SA wrought SS316L seamless tubes and CD wrought SS316L rod bars were included in the investigation. The tubes were 22 mm in diameter and 2 mm in thickness, while the rod bars were 25 mm in diameter. The dimensions of all BMD-manufactured specimens, including thickness, width, and length, were within 10% of the original design after sintering. 

### 2.2. Analytical Characterization

Table 2 presents the elemental composition of the BMD SS316L used in this investigation determined with inductively coupled plasma atomic emission spectroscopy (ICP-AES) analysis. The chemical compositions of SA wrought SS316L and CD wrought SS316L, as given in their material test reports (MTR), are also presented in Table 2. The UNS S31603 nominal chemical composition range is added for comparison. 

The constituent phases were identified via X-ray diffraction (XRD) analysis using a Cobalt K alpha (λ = 0.179 nm) powder diffractometer radiation source operating at 35 kV 40 mA with a LynxEye detector (Bruker, Billerica, MA, USA, D8 Discover). All XRD data were collected within 2θ ranging from 40° to 130°, using a step size of 0.015°, and a time interval of 0.7 s. Content of γ-austenite (FCC) and δ-ferrite (BCC) phases were quantified from the XRD patterns as the area of each crystalline peak over the total area of crystalline peaks. Micro-CT analysis was performed on a 2 × 2 × 2 mm^3^ cut sample using a 3D X-ray microscope with an exposure energy of 140 kV, during an exposure time of 24 h, and at a pixel resolution of 2.2 µm (Zeiss 520 Versa, Oberkochen, Germany). 

Microstructural characterization was conducted on representative samples, which were cut, mounted in cold epoxy resin, manually wet-ground with SiC abrasive papers, and mechanically polished down to 1 µm surface finish. Light optical microscopy (LOM) analysis was conducted on samples chemically etched with a solution containing 100 mL H_2_O, 10 mL HNO_3_, and 100 mL HCl. The concentration of nitric acid and hydrochloric acid was 70% and 32%, respectively. 

Electron backscatter X-ray diffraction (EBSD) analysis was conducted on samples that were polished to a mirror surface finish with 0.02 µm colloidal silica, and then ion-milled for 30 min using a beam voltage of 8 kV at a glancing angle of 4° with full cycle rotational movements (TECHNOORG Linda, Budapest, Hungary, SEMPrep2). Samples were surface-coated with a carbon film 5 µm thick to prevent electrostatic charging. Microstructures were imaged using secondary electron (SE) and backscatter (BS) detectors coupled to a field emission scanning electron microscope (FE-SEM) (TESCAN system, CLARA, Brno, Czech Republic). Elemental composition was mapped with a high-sensitivity Oxford energy-dispersive X-ray spectroscopy (EDS) detection system attached to the FE-SEM. The content of non-metallic inclusions was quantified from EDS elemental maps by dividing the area of oxides or sulfides over the total area of the map, per ASTM E1245 [56]. 

EBSD was conducted with an Oxford symmetry EBSD detector in the FE-SEM on 70° tilted samples, at a working distance of 20 mm, with a beam energy of 28 kV, and a beam current of 1 nA. A clean-up process was applied to the data to assimilate any non- or mis-indexed points, ensuring that a maximum of 10% of the points were modified. Grain boundaries were detected with a threshold misorientation of 10° in conjunction with a minimum of 8 pixels of fractional difference of misorientation variation and a kernel size of 3 by 3. Kernel average misorientation (KAM) maps were used to investigate the presence of local strain in the microstructures. This analysis was conducted using a 3 by 3 kernel size, a square kernel shape, and a maximum misorientation angle of 5°. 

The average grain size was measured as the maximum Feret diameter. The average grain aspect ratio was calculated as the fitted ellipse aspect ratio. Twin content was measured as the fraction length of Σ3 (<111>/60°) boundaries over the total length of γ-austenite (FCC) boundaries. The Schmid factor on the γ-austenite (FCC) phase was measured in the plane/direction {111}<110>. All data acquisition and subsequent post-processing were conducted using the software Aztec v.5.1 and AztecCrystal v.2.1.259, respectively. 

### 2.3. Mechanical Testing

**Figure 1:**
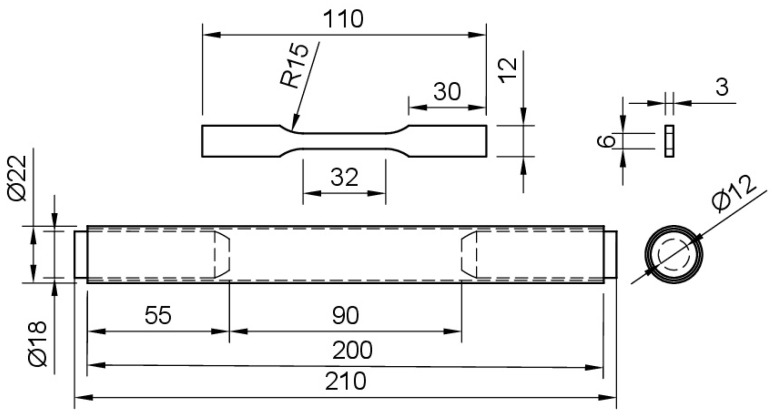
Drawings of the specimens used for tensile testing BMD SS316L and CD wrought SS316L (**top**), and SA wrought SS316L with snug-fitting metallic plugs (**bottom**). Units in millimeters.

Tensile tests were conducted according to ASTM E8 [57]. BMD SS316L and CD wrought SS316L were tested with rectangular specimens, while SA wrought SS316L was tested using tubular specimens with metallic plugs inserted in their ends to ensure a proper grip. All tests were conducted with a 50 kN universal testing machine (UTM, Shimadzu, Kyoto, Japan, AGS-X series). Displacements were measured using an axial extensometer with 25 mm of gauge length and +25 mm of travel length (Epsilon TechCorp, Jackson, WY, USA). The UTM crosshead speed was set to 0.375 mm/min, and the test was stopped once a clear deviation from the initial linear behavior was observed. The actual yield strength (AYS) of each alloy was calculated by intersecting their corresponding stress–strain curves with an 0.2% offset line running parallel to their elastic portion, as per ASTM E8 requirements [57]. Figure 1 shows the geometries and dimensions of the tensile specimens. 

Microhardness was measured on cut samples of untested C-rings prepared similarly to the microstructural characterization procedure that removes the sample preparation induced artifacts. The samples were obtained from the middle of the uppermost curved surface of the C-ring. This test was conducted as per ASTM E384 [58] using a microhardness tester (Duramin-4, Struers, Copenhagen, Denmark), an applied load of 2 Kg (HV2), and a dwell time of 15 s. The bulk density was determined according to ASTM B962 [59] using a density kit coupled to an analytical balance with a readability of 0.001 g and a linearity of ±0.002 g (Mettler-Toledo, ME203, Columbus, OH, USA). The relative bulk porosity content was calculated as the ratio of the measured bulk density and the standard density of UNS S31603 given in ASTM G15 [60]. 

### 2.4. SCC Susceptibility and Crack-Branching

**Figure 2:**
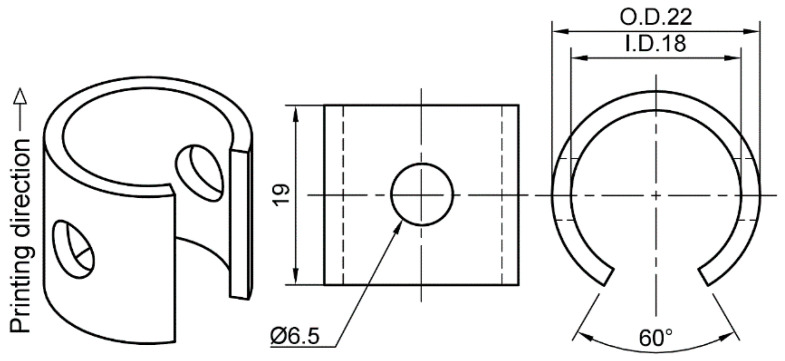
Drawings of the C-ring type specimen used to investigate the SCC susceptibility in the BMD SS316L and its wrought SS316L counterparts. Units in millimeters.

The SCC susceptibility of BMD SS316L and its wrought SS316L counterparts was investigated by exposing C-ring samples to different applied stresses and temperatures. The C-rings were designed following ASTM G38 guidelines [61], see Figure 2. This type of specimen was selected due to its versatility to be elastically deformed at different magnitudes, unlike the U-bent type suggested by ASTM G123 [62]. Duplicate BMD and wrought SS316 C-ring specimens were stressed to 60% and 90% of their AYS to study the effect of stress level on SCC susceptibility. Unstressed C-rings, i.e., 0% AYS, were also tested for comparison and to investigate the possible influence of residual stresses on SCC. Tests were performed in a 25% (by mass) sodium chloride (NaCl) solution, which was acidified to pH 1.5 with phosphoric acid (H_3_PO_4_), as per ASTM G123 [62]. In addition to the standard boiling condition, tests were conducted at different temperatures, i.e., 30, 60, and 80 °C, to define stress–temperature SCC thresholds. 

**Figure 3:**
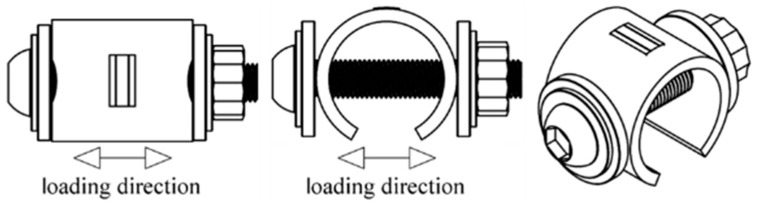
Schematics of the constant-strain setup used to stress the C-rings under different levels of AYS. The circumferential strain gauge is located at the uppermost curved surface of the C-ring.

BMD SS316L C-rings were 3D printed in the vertical direction as shown in Figure 2. All surfaces were wet-ground from 80-grit to 600-grit with SiC abrasive paper, avoiding any excessive removal of material. Subsequently, C-rings were constant-strained to the required level, as per ASTM G38 [61]. The constant-strain setup, which is shown in Figure 3, consisted of two PEEK washers, two M6 titanium flat washers, one M6 titanium socket cap bolt, one M6 titanium flanged lock nut, and a strip of clear PTFE heat shrinkable tube molded to the bolt. The required strain level was obtained by attaching a 0.3 mm circumferential strain gauge (Tokyo Measuring Instruments, Tokyo, Japan, FLAB-03-11-1LJC-F) to the uppermost curved surface at the middle of the C-ring’s arc and width, as shown in Figure 3. Then, the bolt was tightened until the reading in the data logger (Ahlborn, Sayner, WI, USA, Almemo 2590) indicated the required strain value corresponding to 60% and 90% AYS. All traces of the strain gauges and adhesive were manually removed with 600-grit SiC abrasive paper. The electrical insulation between the titanium bolt and the C-ring was verified with a digital multimeter. The C-rings tested at 0% AYS were also prepared, as shown in Figure 3, but no strain was applied in this case. SA wrought SS316L and CD wrought SS316L C-rings were prepared following an identical procedure.

SCC tests were conducted by immersing the C-rings in a series of Erlenmeyer flasks containing 750 mL of solution at the set constant temperatures. Each temperature was monitored regularly with a thermocouple. Duplicate specimens of stressed and unstressed BMD SS316L C-rings and their wrought counterparts were immersed in the solution. Each Erlenmeyer flask contained three different C-rings, i.e., one from each alloy stressed at the same level. The volume of solution per exposed C-ring surface area ratio was 11 mL/cm^2^, which is twice the minimum ratio according to the ASTM G123 standard [62]. All C-rings were standing on their washers to prevent stagnant solution spots at the contact points. C-rings were removed weekly from the solution and inspected for cracks at a magnification of 20× using a LOM. If no cracks were observed, the specimens continued the test in a freshly prepared solution. If cracks were found, cracked specimens were removed from the test and prepared for microscopy analysis. The tests continued for a maximum of six weeks, as per ASTM G123 [62]. The degree of crack-branching was calculated by dividing the total crack length, which includes both the primary and secondary cracks, by the length of the primary crack. LOM images at 10× magnifications were used for this purpose. This approach is consistent with other investigations [52,63]. Size and depth of pits were measured according to the ASTM G46 standard [64].

## 3. Results

### 3.1. Analytical Characterization

**Figure 4:**
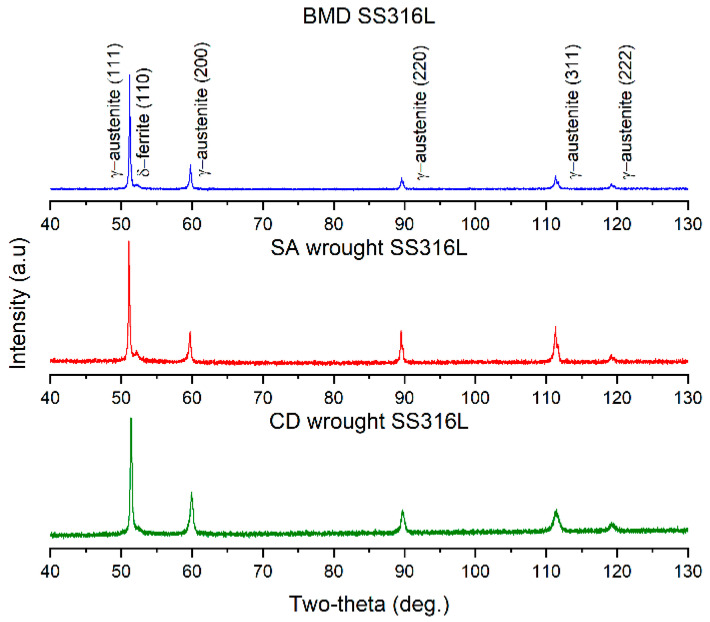
Representative XRD patterns of BMD SS316L, SA wrought SS316L, and CD SS316L showing predominance of the γ-austenitic with small fractions of δ-ferrite.

Representative BMD and wrought SS316 XRD patterns are presented in Figure 4, as indicated. The XRD patterns indicated that all the alloys contained almost entirely γ-austenite (FCC) with a minor presence of δ-ferrite (BCC) phase. The amount of δ-ferrite is summarized in Table 3. Retained δ-ferrite in a relatively low temperature powder-based additively manufactured SS316L can originate from the gas atomization process of the pre-alloyed powder feedstock due to the ferrite-stabilizer effect of Cr, Mo, and Si [41]. Therefore, the small amount of δ-ferrite found in BMD SS316L suggests that its allotropic transformation into the γ-austenite was incomplete during the sintering stage. 

**Figure 5:**
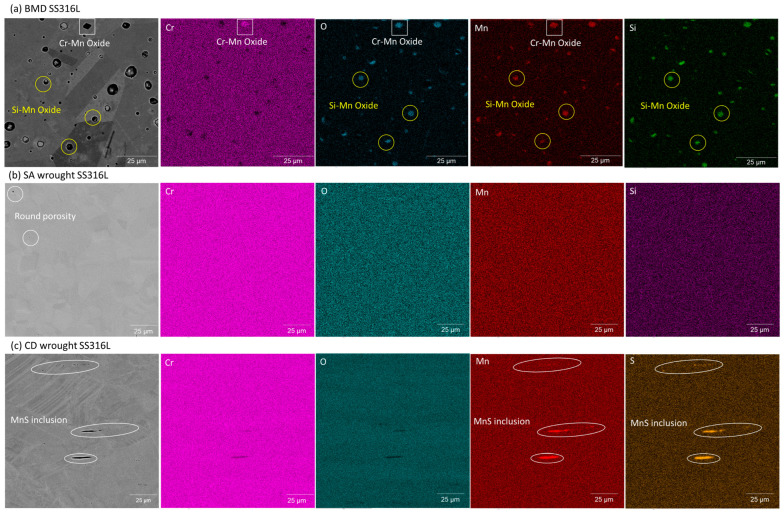
Representative EDS elemental map of (**a**) BMD SS316L showing inclusions rich in O, Si, Mn, and Cr, (**b**) SA wrought SS316L showing slight presence of round pores and lack of non-metallic inclusions, and (**c**) CD wrought SS316L showing elongated MnS inclusions.

SEM-EDS analysis showed no evidence of sensitization, i.e., Cr depletion in the vicinity of the grain boundaries, in any of the SS316L alloys, as illustrated in the elemental map in Figure 5. BMD SS316L contained non-metallic particles rich in O, Si, Mn, and Cr, as seen in Figure 5a, which are inherent to PM and MIM manufacturing processes [65,66,67]. SA wrought SS316L had an almost negligible amount of round pores, and no oxide inclusions were found, Figure 5b. CD wrought SS316L contained manganese sulfide inclusions (MnS), Figure 5c, common in cold-worked austenitic stainless steels [35,68,69]. Table 3 summarizes the content of non-metallic inclusions in BMD SS316L and its wrought counterparts. 

**Figure 6:**
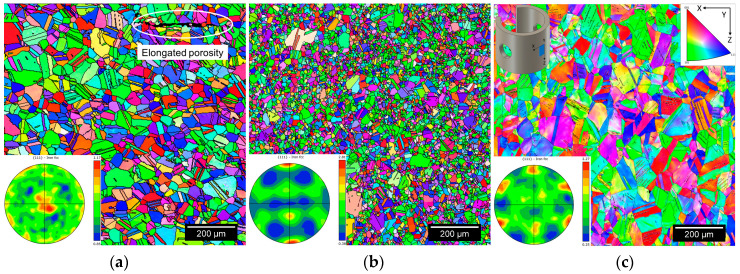
Representative EBSD maps with corresponding {111} pole figures of (**a**) BMD SS316L, (**b**) SA wrought SS316L, and (**c**) CD wrought SS316L C-rings taken from their uppermost curved surfaces.

Figure 6 shows representative inverse pole figure EBSD maps with respect to the build direction (*Y*-axis) of BMD SS316L and its wrought SS316L counterparts. The corresponding BMD SS316L {111} pole figures in Figure 6a show a weakly textured, almost randomly oriented distribution with low intensity (×1.17 random). In comparison, Figure 6b,c shows a slight texture strengthening in wrought SS316L (×2.81 random) and CD wrought SS316 (×2.27 random), as indicated. These texture developments are assumed to be caused by the processing history, which is beyond the scope of this study. Figure 6a also shows an elongated pore in the BMD SS316L sample, perpendicular to its build direction. This type of porosity is inherent to the extruding nature of FDM manufacturing [70,71]. 

**Figure 7:**
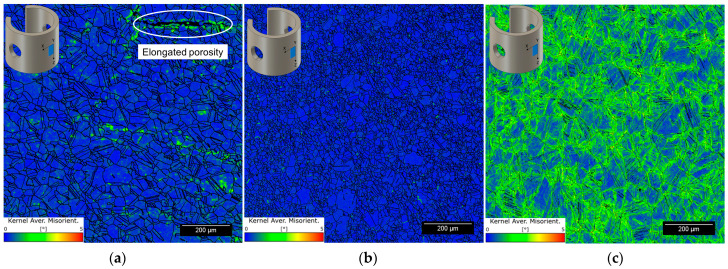
Representative KAM maps with a maximum misorientation angle of 5° in (**a**) BMD SS316L, (**b**) SA wrought SS316L, and (**c**) CD wrought SS316L C-rings taken from their uppermost curved surfaces.

Figure 7 shows the KAM maps of the corresponding EBSD scans in Figure 6, illustrating areas of slight local plastic deformation, i.e., residual stresses, in the microstructure of BMD SS316L. Figure 7b shows negligible local straining in SA wrought SS316L, as opposed to CD wrought SS316L which contained substantial residual stresses, Figure 7c. The distinct degree of residual stresses observed in the wrought materials is caused by their processing conditions [72], which are also beyond the scope of this study. Grain measurements, such as average grain size, aspect ratio, twin boundary content, and Schmid factors are included in Table 3. Further information regarding the influence of the sintering process on the microscopy of the BMD-manufactured SS316L can be found in a previous publication by Santamaria, R., et al. (2021) [9].

### 3.2. Mechanical Testing

**Figure 8:**
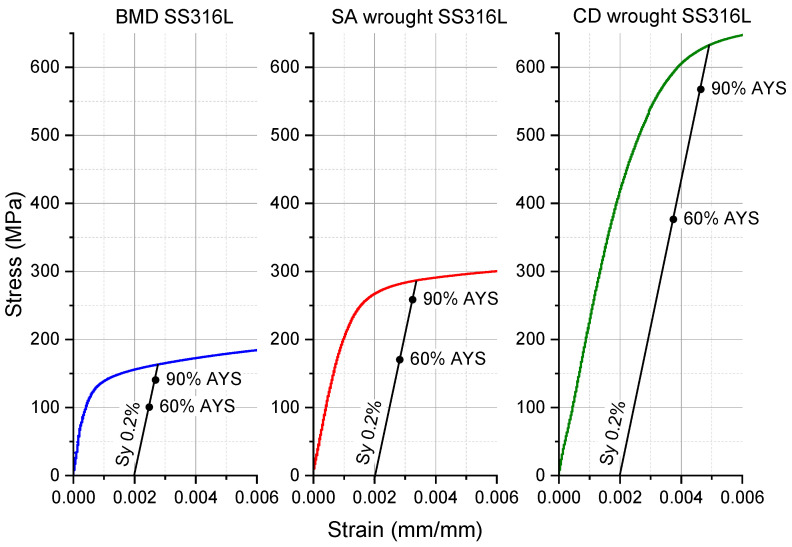
Stress–strain curves within the elastic region of BMD SS316L, SA wrought SS316L, and CD wrought SS316L showing their corresponding 60% and 90% AYS.

Figure 8 presents the elastic regions of the engineering stress–strain curves of BMD SS316L and SA and CD wrought SS316L, indicating their corresponding 60% and 90% AYS values. As shown in Figure 8, CD wrought SS316L had the highest average AYS due to cold working, i.e., 646 ± 8 MPa, followed by the SA wrought SS316L, i.e., 293 ± 6 MPa, and lastly, the BMD SS316L with an AYS of 167 ± 2 MPa. Similar values of AYS in AM sinter-based SS316L have been reported elsewhere [8,73,74,75]. Table 4 summarizes microhardness, bulk density, and relative bulk porosity content. 

Santamaria et al. (2021) [9] conducted a detailed investigation on the impact of the sintering process on the tensile properties and fracture behavior of the BMD-manufactured SS316L. They found that the AM SS316L had a lower yield and tensile strength, caused by its relatively larger grain sizes. However, AM SS316L showed excellent ductility attributed to the abundance of twin boundaries. The AM SS316L fractured in a ductile manner, with spherical dimples uniformly distributed throughout the fracture surface, containing evidence of oxide inclusions. No secondary cracks or parabolic dimples were observed, indicating that the fracture was due to pure tension. Additionally, the necked region exhibited no cup and cone shape, attributed to the tensile flow instability phenomena. 

### 3.3. Pitting and Cracking Susceptibility

**Figure 9:**
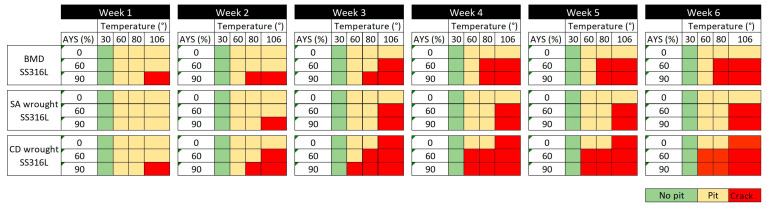
Pitting and cracking susceptibility map of BMD SS316L and its wrought SS316L counterparts at different test conditions over a period of six weeks.

Figure 9 summarizes the susceptibility to pitting and cracking initiation of BMD and SA and CD wrought SS316L at different stress and temperature levels over six weeks, as indicated. In Figure 9, cells colored in green represent no pitting, cells colored in yellow indicate that pitting was observed, and cells colored in red indicate SCC had occurred. At 30 °C, none of the C-rings showed evidence of pitting for the duration of the tests. However, pits were observed in all alloys within the first week when exposed to higher temperatures, i.e., 60 °C and above. In boiling solution (~106 °C), pits quickly (Week 1) transitioned into cracks for BMD and CD wrought SS316L at 90% AYS. The SCC resistance of all alloys decreased with increasing time, stress, and temperature, in agreement with the literature [18,20,33,76]. Figure 9 also shows that SA wrought SS316L had the highest SCC resistance, as indicated by only 2 out of 12 conditions that led to cracking, followed by BMD SS316L with 4 conditions, and finally, CD wrought SS316L with 7 conditions. It is noteworthy to mention that in stressed CD wrought SS316L specimens, all cracks started from the sharp edges of their curved surface, whereas in unstressed specimens, cracks initiated at their flat surface. The difference in crack initiation location was attributed to the residual stresses introduced during the manufacturing process and subsequent machining of the C-rings [76,77,78,79]. 

**Figure 10:**
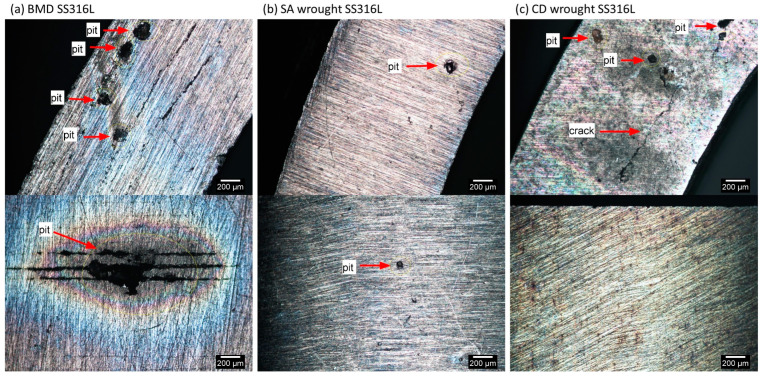
LOM images of unstressed C-rings after one week in boiling solution, showing different sizes of corrosion pits in (**a**) BMD SS316L, (**b**) SA wrought SS316L, and (**c**) CD wrought SS316L. The top images correspond to the C-rings’ flat surfaces, while the curved ones are presented at the bottom.

Figure 10 illustrates the size and distribution of corrosion pits in unstressed BMD and SA and CD wrought SS316L C-rings on their flat and curved surfaces after a week of exposure to the boiling solution. As shown in Figure 10, the printed material had the largest pits, while both wrought counterparts exhibited smaller pits that were similar in size. However, CD wrought SS316L had more pits than the other two alloys. Figure 10 also confirmed that residual stresses were sufficient to cause SCC in the unstressed CD wrought SS316L specimens after a week of immersion in the boiling solution. Table 5 summarizes the average pit size measurements made on unstressed C-rings after one week in boiling solution, as well as the average pit depth in cracked specimens under stress. As seen in Table 5, BMD SS316L had the largest and deepest pits in both measured conditions, while SA wrought had the smallest ones. 

### 3.4. Crack-Branching Susceptibility

**Figure 11:**
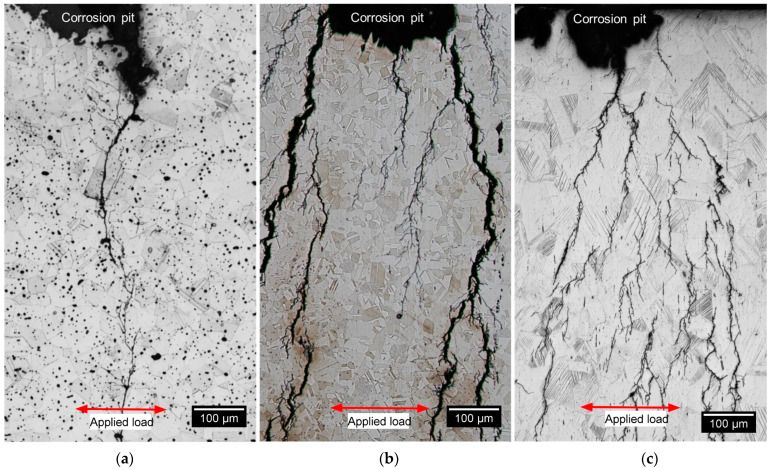
LOM images of etched microstructures in (**a**) BMD SS316L, (**b**) SA wrought SS316L, and (**c**) CD wrought SS316L C-rings after exposure to boiling solution, showing SCC initiated from pits and propagated perpendicular to the applied stress of 90% AYS.

Figure 11 shows transgranular SCC in BMD, SA and CD wrought SS316L after exposure to the boiling solution under a stress of 90% AYS. Identical crack morphology was observed in all specimens regardless of the applied stress and temperature. SCC started from pits and propagated perpendicularly to the applied stress direction, in agreement with the literature [33,76,80,81]. Figure 11 also shows that BMD SS316L had the least amount of crack-branching while both wrought SS316L counterparts cracked in a similar fashion. The calculated crack-branching ratio for BMD and SA and CD wrought SS316L was 1.84 µm/µm, 4.29 µm/µm, and 4.64 µm/µm, respectively. 

**Figure 12:**
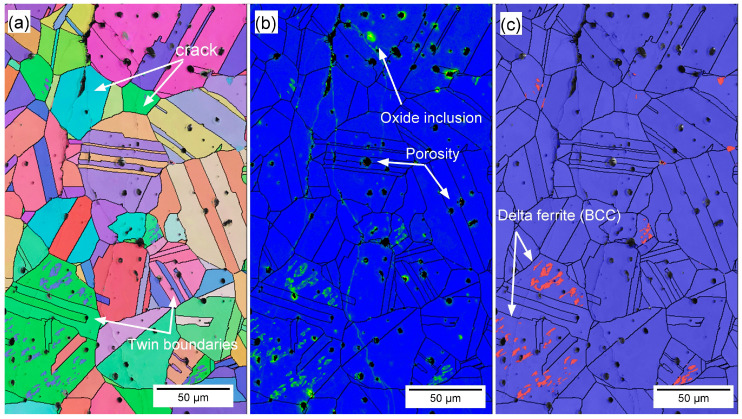
Representative (**a**) EBSD map, (**b**) KAM map, and (**c**) phase map with overlaid band contrast of BMD SS316L, showing transgranular cracking, twin boundaries, oxide inclusions, round porosity, and area of δ-ferrite.

**Figure 13:**
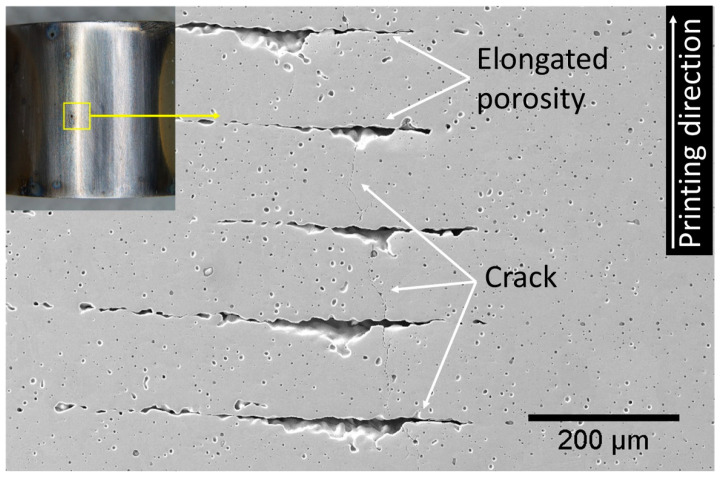
Photographic and SEM image of the curved surface of a BMD SS316L C-ring after exposure to boiling solution under 90% AYS, showing SCC crossing perpendicular to the elongated porosity.

The transgranular nature of SCC in BMD SS316L was also confirmed by the EBSD analysis shown in Figure 12, which include all Euler map, KAM, and phase distributions along with the overlaid band contrast images to facilitate locating crack propagation through the grains. Figure 12 also illustrates some of the characteristic features of the BMD SS316L microstructure, such as twin boundaries, round porosity, oxide inclusions, and δ-ferrite (BCC). Additionally, Figure 13 shows an SEM image of a crack that propagated through elongated pores of a BMD SS316L sample without branching. The lack of branching was attributed to the arresting effect of the pores, which is also visible in the micro-CT scan in Supplementary Video S1.

## 4. Discussion

The results from this study are consistent with the established body of knowledge showing that non-sensitized austenitic stainless steels under tensile stress are susceptible to transgranular SCC when exposed to hot acidic chloride solutions, i.e., 60 °C or above [16,18,20,34]. Additionally, results demonstrated that, when tested under the same conditions, BMD SS316L was more susceptible to SCC initiation than SA wrought SS316L but, given its much lower strength, more resistant than CD wrought SS316L, Figure 9. It is important to note that samples were stressed at a fixed percentage of their AYS. Thus, the actual stress level of CD wrought SS316L was substantially higher at 60% and 90% AYS (i.e., 388 MPa and 581 MPa) than SA wrought (i.e.,176 MPa and 264 MPa) and BMD (i.e., 100 MPa and 150 MPa) samples. Nevertheless, the results are considered valid since the loading conditions represent the reasonable utilization values for the materials in service, where designers take advantage of the higher yield strength of the CD wrought material. Results of unstressed samples also highlighted the influence of residual stresses on SCC susceptibility, with CD wrought samples experiencing SCC cracks after three weeks of exposure to the boiling conditions. 

The manufacturing route influenced SCC morphology. Highly branched cracks are a frequent SCC characteristic of austenitic SS in chloride solutions [33,37,82]. SA and CD wrought samples exhibited the expected branched morphology. In contrast, BMD SS316L showed transgranular cracking with little to no branching, Figure 11. Given that all alloy compositions met the requirements of the UNS S31603 alloy type [36], the different crack morphologies can be attributed to differences in alloy microstructure features such as defects and chemistry-phase-crystallography distributions. 

### 4.1. Susceptibility to SCC Initiation: Pit-to-Crack Transition

The susceptibility map in Figure 9 shows that CD wrought SS316L had the lowest SCC resistance. As discussed above, SCC susceptibility depends on the extent of plastic deformation of the cold-worked condition [35,82,83]. Cold working introduced substantial residual stresses, as shown in the KAM map in Figure 7, promoting SCC nucleation [77]. High-strength SSs, such as CD wrought SS316L, are known to have a low threshold stress intensity factor for SCC (K_I,SCC_), an indication of their low SCC arrest capacity [16,27,31,84]. Due to their lower strength and applied loads, SA wrought and BMD SS316L had improved resistance to SCC initiation. However, BMD exhibited a relatively lower SCC resistance than SA wrought SS316L, especially considering the AM samples’ lower strength. 

The lower SCC resistance of BMD SS316L was attributed to the higher content of microstructural heterogeneities, such as pores and oxide inclusions, Figure 5 and Table 3 and Table 4. These defects serve as preferred stable pit nucleation sites [67,85,86,87,88]. Faster sharp pit propagation, in turn, facilitates the so-called pit-to-crack transition [21,22,23,24,25,26]. The more extensive and deeper pits in BMD SS316L, Figure 10 and Table 5, negatively affected SCC resistance. The SCC resistance of BMD SS316L could be markedly improved by decreasing porosity and oxide inclusions. Strategies to reduce SCC susceptibility include using low-oxygen powder feedstock and prolonging the sintering time to reduce pore size, albeit at the expense of grain growth [65,66,67]. Post-processing steps such as high isostatic pressure (HIP) could also be introduced to close the bulk porosity [5,89,90,91,92]. Lastly, shot-peening could also close surface pores and introduce compressive residual stresses [93,94,95,96]. 

### 4.2. Crack-Branching

The noticeable difference in SCC morphology between wrought and BMD SS316L samples, illustrated in Figure 11, was attributed to the presence of randomly oriented equiaxed grain aggregates—with minimal or no influence of special boundaries—and to a high content of twin boundaries in the BMD microstructure, Figure 6 and Table 3. These features are commonly found in materials processed with sinter-based manufacturing technologies [5,8,9,73,74,75], which act as barriers for crack-branching of transgranular SCC. 

The weakly crystallographic textured microstructure of BMD SS316L resulted in an overall reduction of the Schmid factor, as given in Table 3. The Schmid factor indicates the increased resolved shear stress to initiate the slip across grains [97,98,99,100,101]. Furthermore, the equiaxed grains are crystallographically randomly oriented and comprise a larger amount of twin boundary fractions in BMD SS316L, thus enhancing the resistance to crack propagation via branching in non-localized directions [98,100,101,102,103]. In addition, the higher porosity in BMD SS316L acted as an obstacle to crack-branching. A similar arrestor effect, caused by the blunting of the crack tips, has been reported elsewhere in additively manufactured porous alloys [104,105,106]. The influence of the non-metallic inclusions and retained δ-ferrite (BCC) on the resistance to crack-branching could not be determined since no clear relationship was observed.

## 5. Conclusions

This work determined the SCC behavior of SS316L additively manufactured by sinter-based material extrusion. Tests were conducted in an acidified chloride solution (25 wt% NaCl, pH 1.50) at different stress levels and temperatures to identify SCC thresholds. Results were compared with the SCC response of conventional SA and CD wrought SS316L. Results were supported by a thorough characterization that included LOM, SEM-EDS, EBSD, and micro-CT. The following conclusions were drawn based on the evidence presented above:

1. SCC resistance increased in the following order: SA wrought > BMD > CD wrought SS316L.
2. The sinter-based manufacturing process used to produce BMD SS316L resulted in lower residual stresses and lower strength, contributing to a higher SCC initiation resistance than the highly stressed CD wrought condition.
3. The large grain aggregates, equiaxed grain morphology, weak crystallographic texture, and a large content of twin boundaries decreased the SCC crack-branching of BMD SS316L when compared to SA and CD wrought SS316L.
4. The porosity distribution of BMD SS316L had a mixed impact on its SCC resistance. While these defects facilitated the pit-to-crack transition, they also acted as crack arrestors by blunting the crack tips.

## Figures and Tables

**Table 1:** Summary of parameters used to manufacture all BMD SS316L test specimens.

Printing Parameters		Debinding Parameters	
Extrude line width:	0.5 mm	Debinding time:	15 h
Deposited layer height:	0.15 mm	Debinding temperature:	50 °C
Contour shell thickness:	1.50 mm	Debinding pressure:	Atmospheric
Extrusion nozzle size:	0.40 mm	Sintering parameters	
Extrusion rate:	30 mm/s	Heating rate:	~1.0 °C/min
Extrusion temperature:	175 °C	Thermal debinding temperature:	550 °C
Build plate temperature:	60 °C	Thermal debinding dwell time:	2 h
Sintering scale factors:	X = Y = Z = 1.15	Sintering temperature:	1350 °C
Bulk volume raster pattern:	+45°/−45° each layer	Sintering atmosphere:	Ar > 99.997% vol.
Infill density:	100%	Sintering dwell time:	2 h
Print orientation:	Vertical (Z)	Cooling rate:	Furnace cooling

**Table 2:** Elemental composition in wt% of BMD SS316L, SA wrought SS316L, CD wrought SS316L, and nominal composition of UNS S31603.

Alloy	Source	Fe	C	Cr	Ni	Mo	Si	Mn	P	S
BMD SS316L	ICP-AES	Bal.	0.020	16.3	10.4	2.12	0.61	1.22	0.010	0.010
SA SS316L	MTR	Bal.	0.012	16.1	10.1	2.03	0.46	0.92	0.036	0.002
CD SS316L	MTR	Bal	0.019	16.7	10.1	2.03	0.41	1.72	0.024	0.025
UNS S31603	ASTM A213 [36]	Bal.	Max. 0.035	16.0 18.0	10.0 14.0	2.00 3.00	Max. 1.00	Max. 2.00	Max. 0.045	Max. 0.030

**Table 3:** Content of non-metallic inclusions, δ-ferrite (BCC) phase, and grain size measurements of BMD SS316L, SA wrought SS316L, and CD wrought SS316L.

Alloy	Non-Metallic Inclusions (%)	δ-Ferrite Phase (%)	Average Grain Size (µm)	Aspect Ratio	Twin Boundaries (%)	Schmid Factor {111}<110>
BMD SS316L	3.23	6.09	40.8 ± 23.8	3.1 ± 2.4	53.2	0.69
SA SS316L	0.01	7.86	16.2 ± 8.5	2.2 ± 1.3	45.5	0.94
CD SS316L	0.39	0.95	43.5 ± 33.6	3.9 ± 3.6	39.4	0.96

**Table 4:** Average mechanical properties of BMD SS316L and its wrought counterparts.

Alloy	AYS (MPa)	Microhardness (HV2)	Bulk Density (g/cm^3^)	Relative Bulk Porosity (%)
BMD SS316L	167 ± 2	117.1 ± 3.2	7.564 ± 0.013	5.21
SA SS316L	293 ± 6	163.9 ± 2.5	7.935 ± 0.025	0.57
CD SS316L	646 ± 8	277.3 ± 3.2	7.953 ± 0.027	0.35

**Table 5:** Average pit size and pit depth measurements made on stressed and unstressed BMD SS316L C-rings and wrought counterparts.

Alloy	Unstressed C-Ring (0%AYS) in Boiling Solution after 1 Week		Stressed C-Ring (90%AYS) in Boiling Solution after Cracking	
	Pit Size (µm) in Flat Surface	Pit Size (µm) in Curved Surface	Pit Size (µm)	Pit Depth (µm)
BMDSS3 16L	112 ± 117	89 ± 154	406 ± 359	190 ± 135
SA SS316L	31 ± 27	28 ± 16	213 ± 178	58 ± 27
CD SS316L	88 ± 61	27 ± 11	205 ± 118	130 ± 84

## Data Availability

This article contains all the data that was generated and is presented in the form of Figures, Tables, or Supplementary Materials.

## Supplementary Materials

The following supporting information can be downloaded at: https://www.mdpi.com/article/10.3390/ma16114006/s1, Video S1: Micro-CT scan of BMD SS316L with respect to its build direction (Z) showing the propagation of the secondary cracks being arrested by the elongated porosity.
